# Signaling pathways in the regulation of cytokine release syndrome in human diseases and intervention therapy

**DOI:** 10.1038/s41392-021-00764-4

**Published:** 2021-10-20

**Authors:** Xia Li, Mi Shao, Xiangjun Zeng, Pengxu Qian, He Huang

**Affiliations:** 1 Bone Marrow Transplantation Center, The First Affiliated Hospital, Zhejiang University School of Medicine, Hangzhou, People’s Republic of China; 2 Liangzhu Laboratory, Zhejiang University Medical Center, 1369 West Wenyi Road, Hangzhou, 311121 People’s Republic of China; 3 Institute of Hematology, Zhejiang University, Hangzhou, Zhejiang People’s Republic of China; 4 Zhejiang Province Engineering Laboratory for Stem Cell and Immunity Therapy, Hangzhou, Zhejiang People’s Republic of China; 5 Center of Stem Cell and Regenerative Medicine, Zhejiang University School of Medicine, Hangzhou, People’s Republic of China

**Keywords:** Infectious diseases, Immunotherapy, Immunological disorders

## Abstract

Cytokine release syndrome (CRS) embodies a mixture of clinical manifestations, including elevated circulating cytokine levels, acute systemic inflammatory symptoms and secondary organ dysfunction, which was first described in the context of acute graft-versus-host disease after allogeneic hematopoietic stem-cell transplantation and was later observed in pandemics of influenza, SARS-CoV and COVID-19, immunotherapy of tumor, after chimeric antigen receptor T (CAR-T) therapy, and in monogenic disorders and autoimmune diseases. Particularly, severe CRS is a very significant and life-threatening complication, which is clinically characterized by persistent high fever, hyperinflammation, and severe organ dysfunction. However, CRS is a double-edged sword, which may be both helpful in controlling tumors/viruses/infections and harmful to the host. Although a high incidence and high levels of cytokines are features of CRS, the detailed kinetics and specific mechanisms of CRS in human diseases and intervention therapy remain unclear. In the present review, we have summarized the most recent advances related to the clinical features and management of CRS as well as cutting-edge technologies to elucidate the mechanisms of CRS. Considering that CRS is the major adverse event in human diseases and intervention therapy, our review delineates the characteristics, kinetics, signaling pathways, and potential mechanisms of CRS, which shows its clinical relevance for achieving both favorable efficacy and low toxicity.

## Introduction

Cytokine release syndrome (CRS) is characterized by a mixture of clinical manifestations, including elevated circulating cytokine levels, acute systemic inflammatory symptoms, and secondary organ dysfunction, while severe CRS is a very significant and life-threatening complication. Given the lack of a unified and widely accepted definition of CRS, Fajgenbaum and his colleagues proposed the following three criteria to identify CRS: (1) elevated circulating cytokine levels, (2) acute systemic inflammatory symptoms, and (3) secondary organ dysfunction (often renal, hepatic, or pulmonary) due to inflammation beyond that which could be attributed to a normal response to a pathogen (if a pathogen is present) or any cytokine-driven organ dysfunction (if no pathogen is present).^1^ However, CRS is a double-edged sword, which may be both helpful in controlling tumors/viruses/infections and harmful to the host.

It has important clinical relevance to acknowledge that CRS facilitates disease control and may be harmful to the host. To better understand the cause of CRS, the history and general characteristic of CRS were initially introduced. Then, we summarized clinical features and risk factors of CRS caused by iatrogenic factors, pathogenic factors, monogenic disorders, and autoimmune diseases. The cellular and molecular mechanisms of CRS among these human diseases were further highlighted. Finally, CRS models, cutting-edge technologies, management of CRS, and future direction were also discussed.

## The history and general characteristics of CRS

**Fig. 1:**
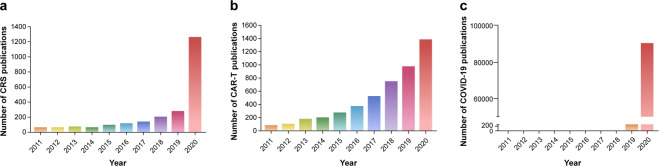
The increasing number of publications searched by the key words ‘cytokine release syndrome’ (**a**), ‘chimeric antigen receptor T (CAR-T) cell’ (**b**) or ‘COVID-19’ (**c**) in PubMed (https://pubmed.ncbi.nlm.nih.gov) in recent decades

In 1993, Ferrara et al. coined the term ‘cytokine storm’ to describe the clinical manifestations of acute graft-versus-host disease after allogeneic hematopoietic stem-cell transplantation.^2^ Later, cytokine storm was also known as cytokine release syndrome (CRS) and reported in the influenza pandemic^3^ and severe acute respiratory syndrome coronavirus (SARS-CoV).^4^ In addition, chimeric antigen receptor T cell (CAR-T cell) immunotherapy was the fourth pillar in cancer therapeutics,^5–11^ in which CRS was the most common clinical complication and was first reported in 2010.^12^ Meanwhile, coronavirus disease 2019 (COVID-19), which is characterized by severe respiratory illness in humans, is also accompanied by CRS.^13–15^ Furthermore, the significant boom in the publication of studies with the key words ‘cytokine release syndrome’, ‘chimeric antigen receptor T cell’, or ‘COVID-19’ in PubMed (https://pubmed.ncbi.nlm.nih.gov/) in recent decades suggests that CRS, CAR-T, and COVID-19 are important issues in the fields of cancer, immunotherapy, and viral infection (Fig. 1). Meanwhile, CRS phenomenon can be also observed in other human diseases, such as monogenic disorders^16^ and autoimmune diseases.^17^

In general, CRS caused by various factors involves a mixture of common elevated cytokines, including tumor necrosis factor (TNF)-α, interleukin (IL)−1, IL-6, and interferon gamma (IFN-γ). To further explore the CRS-related cytokine functions, we summarized 12 cytokines and divided them into three groups: antitumor effectors (granzyme B (GZMB), IFN-γ, macrophage inflammatory protein (MIP)-1α, and TNF-α),^18–22^ stimulatory and regulatory cytokines (GM-CSF, IL-2, MCP-1, NO, and IL-15),^23–28^ and inflammatory cytokines (IL-1, IL-6, and IL-17A).^27,29,30^ Their sources, functions and related studies are summarized in Table 1.

**Table 1:** The sources and functions of CRS-related cytokines in different CRS studies

Cytokines	Sources	Functions	Reference
Antitumor effector cytokines			
GZMB	Cytotoxic T lymphocytes, NK cells	Antitumor, proapoptotic, and antiviral activities	Ma et al. (2020);^18^ Gust et al. (2019)^19^
IFN-γ	Th1 cells, NK cells, CD8 T cells	Activates macrophages, supports Th1 differentiation, antiproliferative activities, weakens antiviral activity	Zhang et al. (2019)^20^
TNF-α	Monocytes, macrophages, activated T cells, neutrophils	Strong mediator of inflammatory and immune functions, regulates cell growth and differentiation	Zhang et al. (2018)^21^
MIP-1α	Eosinophils	Attracts leukocytes	Xue et al. (2017)^22^
Stimulatory and regulatory cytokines			
GM-CSF	Bone marrow stromal cells, macrophages	Essential for the growth and differentiation of neutrophils	Sachdeva et al. (2019);^23^ Sterner et al. (2019)^24^
IL-2	Activated T cells	Stimulates the proliferation and differentiation of T and B cells, activates NK cells	Golumba-Nagy et al. (2018)^25^
MCP-1	Monocytes, macrophages, tumor cells	Stimulates the motility of T cells, NK cells and basophils; induces the recruitment and activation of monocytes and macrophages during inflammation	Hirayama et al. (2019)^26^
NO	T cells, macrophages, fibroblasts, endothelial cells	Triggers apoptosis	Giavridis et al. (2018)^27^
IL-15	Primarily dendritic cells, cells of the monocyte lineage	Stimulates the proliferation and development of NK cells and T cells	Atilla et al. (2020);^194^ Giuffrida et al. (2020)^28^
Inflammatory cytokines			
IL-1α/IL-1β	Monocytes, macrophages, dendritic cells, endothelial cells, fibroblasts, adipocytes	Induction of local inflammation and systemic effects such as fever, the acute-phase response and stimulation of neutrophil production	Norelli et al. (2018);^29^ Giavridis et al. (2018)^27^
IL-6	T cells, B cells, macrophages, bone marrow stromal cells, fibroblasts	Regulates B and T cell functions, effects on hematopoiesis in vivo, induces inflammation and the acute-phase response	Norelli et al. (2018)^29^
IL-17α	CD4, CD8, and γδ T cells; NK cells	Promotes inflammation by increasing the production of pro-inflammatory cytokines and chemokines that attract monocytes and neutrophils	Rossi et al. (2018)^30^

## CRS in iatrogenic diseases

### CRS in CAR-T-cell therapy

#### Clinical features

**Fig. 2:**
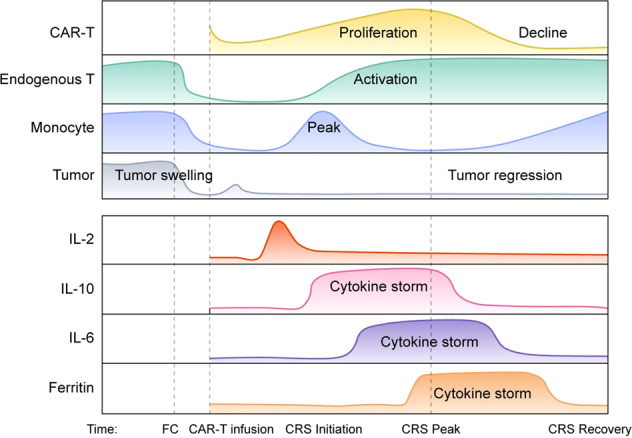
The in vivo kinetics of cell counts and cytokine levels in the serum during the CRS process after CAR-T-cell therapy. Patients were treated with tocilizumab or corticosteroids when CRS reached at grade 3–4

The clinical manifestations of CRS are characterized by high fever, hypotension, hypoxia, capillary leakage, coagulopathy, respiratory distress and severe organ dysfunction.^31^ Severe complications, including liver transaminitis and renal insufficiency, are life-threatening and frequently require ICU care with vasopressors and/or ventilation support.^32,33^ To better understand the dynamic changes in CRS, the present review artificially divided CRS into three stages based on the general changing dynamics of CRS-related cytokine levels and CAR-T-cell counts: CRS initiation stage, CRS peak stage, and CRS recovery stage. In the CRS initiation stage, the levels of serum TNF-α, IL-1, IL-2, IL-10, and C-reactive protein (CRP) first increase. Then, temperature and the levels of ferritin, IFN-γ, and IL-6 are upregulated from the CRS initiation stage to the peak stage. During the CRS recovery stage, IL-4 and IL-17α levels are increased in turn, while the levels of the other above-mentioned cytokines gradually decline (Fig. 2).

To explore the distribution of different CRS grades, in 2017, Hay et al. reported data for 133 adult patients who received CD19 CAR-T cells.^34^ According to the consensus classification criteria of CRS grade proposed by Lee and his colleagues,^35^ they found that CRS developed in 70% patients, including 62.5% with grade 1~3 CRS and 7.6% with grade 4~5 CRS. In another study, Curran et al. found that 16% (4 of 25) of patients with relapsed/refractory (r/r) B-cell acute lymphoblastic leukemia (ALL) experienced severe CRS.^36^ The percentages of CRS of different grades in different diseases and their classification criteria are also summarized in Table 2.^8,34,37–45^ It is worth noting that the risk of severe CRS in B-cell malignancies is the highest for ALL, lymphoma, and multiple myeloma.

**Table 2:** Studies on the incidence of CRS of different grades and the classification criteria

Disease/Target antigen	Costimulatory domain	CRS classification criteria	CRS percentage (severe rate)	Reference
ALL				
CD19	4-1BB	Consensus criteria and NCI CTCAE v4.03	70% (G3–5: 12%)	Hay et al. (2017)^34^
CD19	CD28	NCI consensus CRS grading system and NCI CTCAE v4.03	80% (G3-4: 16%)	Curran et al. (2019)^36^
CD19	CD28	Modified criteria of Lee and colleagues^35^ and NCI CTCAE v4.02	76% (G3-4: 28%)	Lee et al. (2015)^37^
CD19	4-1BB		77% (G3-4: 47%)	Maude et al. (2018)^38^
CD19	CD28	MSKCC CRS grading system and NCI CTCAE v4.03	85% (G3-4: 26%)	Park et al. (2018)^44^
CD19	4-1BB	Modified criteria of Lee and colleagues and NCI CTCAE v4.03	66.7% (G3-4: 40%)	Hu et al. (2017)^45^
CD22	4-1BB		76%	Fry et al. (2018)^39^
Lymphoma				
CD19	CD28	Modified criteria of Lee and colleagues and NCI CTCAE v4.03	92% (G3-4: 11%)	Locke et al. (2019)^40^
CD19	4-1BB	University of Pennsylvania grading scale and NCI CTCAE v4.03	58% (G3-4: 22%)	Schuster et al. (2019)^8^
Multiple myeloma				
BCMA	4-1BB	Modified criteria of Lee and colleagues and NCI CTCAE v4.03	90% (G3-4: 7%)	Zhao et al. (2018)^195^
BCMA	4-1BB	Modified criteria of Lee and colleagues and NCI CTCAE v4.03	76% (G3-4: 6%)	Raje et al. (2019)^42^
CD19 & BCMA	4-BB & 4-1BB	Modified criteria of Lee and colleagues and NCI CTCAE v4.03	90% (G3-4: 5%)	Yan et al. (2019)^43^
CLL				
CD19	4-1BB	Consensus criteria and NCI CTCAE v4.03	70% (G3-5: 12%)	Hay et al. (2017)^34^

CRS grading is performed using the classification proposed by Lee and his colleagues, while grading of organ toxicities is performed according to Common Terminology Criteria for Adverse Events (CTCAE) Version 4.03

#### Risk factors

Current studies suggest that the severity of CRS is associated with tumor burden (the number of malignant bone marrow cells), basic characteristics of patients (age, gut microflora*,* etc.), and quality/quantity of CAR-T cells (including the construct, infusion dose and amplification ability).

#### Tumor burden

In general, CRS is more frequent and severe in patients with a high tumor burden. The tumor burden may not only determine the peak number of CAR-T cells but also affect the time course of CAR-T-cell proliferation. However, Curran et al. concluded that the dose intensity of conditioning chemotherapy and the minimal pretreatment disease burden positively impacted the response without increasing toxicity.^36^ These phenomena may occur because fludarabine/cyclophosphamide (FC)-mediated lymphodepletion provides more ‘space’ for CAR-T-cell proliferation, reducing competition with other lymphocytes for growth factors and removing inhibitory immune cells.^46^

#### Basic characteristics in patients

In addition to the tumor burden, patient immune status should also be considered for CAR-T-cell therapy and CRS control. Regarding patient aging-related factors, Zettler et al. reviewed a total of 804 cases, which included 471 young patients (<65 years old) and 333 aged patients (>65 years old).^47^ CRS was the most common adverse event reported in both young and aged groups. Although CRS severity was not significantly different, some individual clinical features of CRS, including pyrexia, tachycardia, and thrombocytopenia, were significantly more common in the younger age group. For gut microflora related to individual patient immune status, Kale et al. demonstrated that ‘small’ intestinal immunopathology played a ‘big’ role in lethal CRS in a mouse model.^48^ Furthermore, they found that IFN-γ-JAK/STAT-driven pathways and IL-17α deficiency contributed to lethal small intestinal immunopathology in T cell-driven CRS.

#### Quality and quantity of CAR-T cells

CAR-T cells themselves are also important in CRS. In regard to CAR-T-cell constructs, our group demonstrated that dual-targeted CD19/CD22 CAR-T-cell therapy had a lower incidence of CRS than single-target CD19 CAR-T-cell therapy, especially in severe CRS (≥3 grade CRS).^49^ Additionally, Magnani et al. reported that sleeping beauty-engineered CAR-T cells achieved activity without severe toxicities, with two grade 1 CRS cases and one grade 2 CRS case occurring in 13 patients with B-cell ALL.^50^ On the other hand, the CD4^+^/CD8^+^ ratio of CAR-T-cell infusion products (IPs)^51^ and the dose of administered CAR-T cells^52^ are always positively correlated with the treatment effect and CRS severity. Furthermore, the CAR-T-cell infusion strategy is also a factor to consider. In 2020, Frey et al. demonstrated that compared with a single infusion, fractionated dosing of CAR-T cells with intrapatient dose modification (CAR-T-cell infusion: day 1, 10%; day 2, 30%; and day 3, 60%) optimized safety (low and manageable CRS) without compromising efficacy (higher survival rate) in adults with ALL.^53^

#### CRS in other immune therapy

CRS is not restricted to CAR-T-cell therapy and is also associated with other monoclonal antibody (mAb) treatment. These antibodies include: muromonab (OKT3), rituximab, TGN1412, CP-870,893, blinatumomab, and TDB.

OKT3, an anti-CD3 antibody mainly indicated for the treatment of acute organ transplant rejection, may also lead to severe CRS with increased serum levels of cytokines (e.g., TNF-α, IL-2, IL-6, and IFN-γ).^54,55^ Rituximab, a chimeric anti-CD20 mAb, is widely used to treat hematological malignancies, including CD20-positive diffuse large B-cell lymphoma and^56^ and chronic lymphocytic leukemia.^57^ Severe CRS has occurred in patients receiving rituximab, who had severe dyspnea, fever and blood pressure changes. Cytokine of TNF-α, IL-6, and IFN-γ reached the peak at 2 h after rituximab infusion in chronic lymphocytic leukemia (CLL) patients.^58^ Besides, TGN1412, a superagonist anti-CD28 humanized mAb, can directly stimulate T cells and treatment of healthy volunteers with TGN1412 led to severe CRS and increased disseminated intravascular coagulation (DIC).^59^

On the other hand, the Kantarjian’s group demonstrated that single-agent blinatumomab (a bispecific antibody that binds to CD19 tumor cells and CD3 T cells) showed antileukemia activity in adult patients with relapsed or refractory B-precursor ALL, in which 2% patients had grade 3 CRS.^60^ In 2019, Li et al. found that CRS was also induced after the treatment of anti-HER2/CD3 T cell-dependent bispecific (TDB) antibody.^61^ It is worth noting that systemic serum expression of TNF-α, IL-2, and IL-6 was substantially increased only at 2 h after treatment. Furthermore, TNF-α blockade can prevent macrophage activation without affecting T cell killing capacity and effectively mitigates CRS in anti-CD3 bispecific antibody therapy. In addition, immune checkpoint inhibitors with nivolumab- (anti-programmed cell death-1 mAb) and ipilimumab- (anti-cytotoxic T lymphocyte antigen-4 mAb) induced CRS were also reported in 2021, in which CRP, aspartate aminotransferase, alanine aminotransaminase, and D-dimer were significantly increased after nivolumab and ipilimumab infusion.^62^ At last, other mAbs^63–66^ most commonly associated with CRS are also listed in Table 3.

**Table 3:** Drugs triggered cytokine release syndrome

Antibody	Antigen	Driven cytokine and approximate time of CRS appearance, and Ref.
Muromonab	CD3	TNF-α, IFN-γ, IL-2, and IL-6; 2~4 h after infusion (Yan et al., 2019)^55^
Rituximab	CD20	TNF-α, IFN-γ, and IL-6; 2 h after infusion (Byrd et al., 2001)^58^
TGN1412	CD28	TNF-α, IFN-γ, IL-8, IL-10, and DIC; 1.5 h after infusion (Suntharalingam et al., 2006)^59^
CP-870,893	CD40	TNF-α and IL-6; minutes to hours after infusion (Vonderheide et al., 2007;^64^ and Nowak et al., 2015)^63^
Alemtuzumab	CD52	TNF-α, IFN-γ, and IL-6; 2~4 h after infusion (Ferrajoli et al., 2001;^65^ and Moreau et al., 1996)^66^
Blinatumomab	CD3 & CD19	TNF-α and IL-6; no time information (Topp et al., 2015)^60^
TDB	CD3 & HER2	TNF-α, IL-1, IL-2, and IL-6; 2 h after infusion (Li et al., 2019)^61^
Nivolumab & ipilimumab	PD1 & CTLA4	CRP, aspartate aminotransferase, alanine aminotransaminase, D-dimer; minutes to day 2 after infusion (Urasaki et al., 2021)^62^

## CRS in pathogenic diseases

### CRS in COVID-19

#### Clinical features

CRS is observed in infections and a significant part of the pathogen-related diseases, including bacterial sepsis, HHV-8-associated MCD, and COVID-19. Coronavirus disease 2019 (COVID-19), which is caused by SARS-CoV-2, has become one of the worst pandemics of 2020 and has caused a large number of deaths.^67^ COVID-19 is characterized by heterogeneous symptoms ranging from mild fatigue to life-threatening pneumonia, CRS syndrome, and multi-organ failure.^1^ The laboratory features of COVID-19 include lymphocytopenia, leukopenia, and thrombocytopenia.^68,69^

The elevated cytokines in the serum of patients with COVID-19 include IL-1β, IL-6, IP-10, TNF, IFN-γ, MIP 1α/1β, and VEGF.^70,71^ Furthermore, patients with severe COVID-19 may have significantly higher levels of inflammatory parameters, including CRP, ferritin, D-dimer, and pro-inflammatory cytokines (such as TNF-α, GM-CSF, IL-6, IP-10, MCP-1, and MCP-1a), than patients with mild COVID-19, which demonstrated the presence of a CRS in severe affected patients. Higher IL-6 levels (hyperinflammation) and tissue damage are strongly associated with shorter survival in COVID-19.^72,73^ Wang et al.^74^ found that the increase in IL-6 occurred 1–2 days prior to the decreases in CD8 and CD4 T cells. Liu et al.^75^ observed that CD4 and CD8 T-cell numbers dropped to their lowest levels after 4–6 days of illness, whereas IL-10, IL-2, TNF, and other cytokines reached peak levels.

#### Risk factors

Understanding he possible intricate risk factors associated with COVID-19 severity is helpful in identifying those patients who are at a high risk and require prioritized management to prevent disease progression and severe adverse outcomes. In this section, the severity of the COVID-19 would be discussed in terms of viral load and basic characteristics in patients (age, sex, and underlying diseases).

#### Viral load

Viral load varies greatly among different populations and is closely related to the course of disease.^76^ The cycle threshold values of severe COVID-19 patients were significantly lower than mild COVID-19 patients, and the mean viral load of severe patients was much higher than mild COVID-19 patients.^77^ The kinetics of viral load and exposure to large numbers of infective SARS-CoV-2 were highly predictive markers of severe outcomes in older patients.^78,79^ In addition, Lieberman et al. reported that variability in the antiviral response in males and elderly patients with COVID-19 was dependent on viral load and infection time.^80^

#### Basic characteristics in patients

Higher severity was found to be associated with older age^81^ and male sex.^82^ Data from 79,394 confirmed COVID-19 patients in China showed that those aged below 30 and above 59 years were 0.6 and 5.1 times more likely to die than patients aged 30–59 years, respectively.^83^ Biological age, which was comprised of chronological age and nine clinical chemistry biomarkers, was an optimal predictor of disease severity.^84^

In addition to age and sex, preexisting comorbidities, such as hypertension, diabetes and obesity, are also external risk factors for the survival of critically ill patients with COVID-19. Hypertension, the most frequent comorbidity, was an independent risk factor for assessing the severity of COVID-19,^85^ while a history of chronic obstructive pulmonary disease, hypercholesterolemia, and diabetes was also independently associated with mortality.^86^

#### CRS in other pathogenic diseases

Infectious diseases are a leading cause of death in the whole world. The triggering of the inflammatory response, especially through cytokine production, is essential for the elimination of pathogens during infection. However, uncontrolled cytokine production would result in a CRS. Here, we summarized infection-associated CRS into two common categories: bacterial infections and viral infections.

The involvement of CRS and subsequent immunoparalysis has been reported in the development of bacterial infections with leptospirosis,^87^ streptococcus,^88^ and staphylococcus.^89^ Disseminated bacterial infections can lead to fever, cell death, coagulopathies, and multi-organ dysfunction by producing many cytokines and inducing CRS. For the cellular mechanisms, macrophages are a major cell type driving the CRS during infection. In the development of sepsis, activated macrophages release a great number of pro-inflammatory cytokines and chemokines, including IL-1α, IL-1β, IL-6, and TNF-α, and initiate an inflammatory response.^90^ Certain bacteria can lead to polyclonal activation of T cells, cytokine production, and toxic shock syndrome by producing super antigens that cross-link the major histocompatibility complex and T-cell receptors. For the molecular mechanisms, microbial pathogen-associated molecular patterns (PAMPs) will be recognized by the pattern recognition receptors (PRRs), which are expressed on the surface of innate immune cells.^91^ The PAMPs/PRR triggers an inflammatory cascade by activating multiple signaling modules, including NF-κB and AP-1 transcription factors,^92^ which in turn regulate the expression of cytokines, PGs, and NO.

CRS was also observed in patients infected with influenza virus and coronavirus, such as H1N1, H1N5, SARS-CoV, and MERS-CoV.^93^ Serum levels of IL-8, IL-9, IL-17, IL-6, IL-15, TNF-α, and IL-10 were increased in H1N1 influenza virus infection, while IL-8, IP-10, MCP-1, MIP-1, and CXCL-9 were elevated in H5N1 influenza virus infection.^94^ Meanwhile, the release of IL-6 and IL-1β from other immune cells promotes recruitment of T cells and neutrophils.^95^ In addition, patients infected with EBV or cytomegalovirus may have accompanying perforin functional defects, which leads to defective clearance of antigen-bearing dendritic cells and prolonged engagement between lymphocytes and antigen-presenting cells.^96^ These further resulted in continuous activation and proliferation of T cells and macrophages, and an autocrine loop of pro-inflammatory cytokines.^97^ For the molecular mechanisms during the virus infection, both PAMPs and damage-associated molecular patterns (DAMPs) participate in innate immune responses through the cascade amplification of IFN. Downstream production of IFN promotes intracellular antiviral defenses in neighboring epithelial cells which may limit viral dissemination.

#### CRS in monogenic and autoimmune diseases

CRS phenomena can also be observed in other human diseases, such as monogenic disorders and autoimmune diseases.

#### CRS in monogenic disorders

Recent years have witnessed a broad explosion of new autoinflammatory disorders. The monogenic autoinflammatory diseases are spontaneous or minimally triggered immune activation due to either loss-of-function mutations in genes that suppress inflammation or gain-of-function mutations in genes that propagate inflammation. According to the underlying mechanisms, monogenic autoinflammatory diseases are mainly divided into inflammasome/IL-1 diseases, the interferonopathies, and the NF-κB/TNF disorders.^98^

Inflammasome/IL-1 diseases include familial Mediterranean fever (with MEFV gene disorder), familial cold autoinflammatory syndrome (with NLRP3 disorder), autoinflammation with infantile enterocolitis (with NLRC4 gene disorder), and deficiency of IL-1 receptor antagonist (with L1RN gene disorder), and so on. As a typical inflammasome/IL-1 monogenic disorder, hemophagocytic lymphohistiocytosis (HLH) is characterized by uncontrolled activation of NK cells, cytotoxic T lymphocytes and macrophages, leading to immune-mediated injury of multiple organ systems.^16,99^ Clinically, patients with HLH are characterized by fever, hepatosplenomegaly, cytopenia, and activated macrophages in hemopoietic organs.^100^ The pathophysiology of HLH is that enhanced antigen presentation and repeated IFN-γ-dependent stimulation of Toll-like receptors lead to the uncontrolled activation of antigen-presenting cells and T cells and produce an exaggerated inflammatory response (such as IL-1, IL-4, IL-6, IL-8, IL-10, IL-18, IFN-γ, and TNF-α).^100^ Therefore, medications to block the cytokine network achieved considerable efficacy for controlling HLH. Glucocorticoids, cyclosporine, etoposide, anti-thymocyte globulin, and cytotoxic chemotherapies are effective in controlling both primary and secondary HLH by suppressing or eliminating T cells and other immune cells.^101^ In addition, anti-IL-1 antibodies, anti-IL-6 antibodies, JAK1 and JAK2 inhibitors, and anti-IFN-γ antibodies are also effective in blocking specific cytokines and controlling HLH.^1^

Interferonopathies include Singleton-Merten syndrome (with IFIH1 or DDX58a gene disorder) and STING-associated vasculopathy of infancy (with TMEM137 gene disorder). NF-κB/TNF disorders include haploinsufficiency of A20 (with TNFAIP3 gene disorder), OTULIN-related autoinflammatory syndrome (with OTULIN gene disorder), Blau syndrome (with NOD2 gene disorder), TNF receptor-associated periodic syndrome (with TNFRSF1A gene disorder), and deficiency of adenosine deaminase 2 (with ADA2 gene disorder). Moreover, Janus kinase inhibitor and TNF inhibitor are the main targeted therapy for the above two kinds of monogenic autoinflammatory diseases, respectively.

#### CRS in autoimmune diseases

Besides autoinflammatory diseases, autoimmune diseases are another axis of immune dysfunction which mistakenly target autologous tissues as if they were foreign. Autoimmune diseases would lead to seemingly unprovoked inflammation and CRS but do not show any signs of infection or autoimmunity. Systemic lupus erythematosus (SLE), a classical kind of autoimmune disease, is characterized by exaggerated B-cell and T-cell responses and loss of immune tolerance against self-antigens, leading to variable clinical symptoms, including low-grade fever, fatigue, weight loss and other target organ manifestations, which are accompanied by elevated levels of autoimmune antibodies and a series of cytokines.^102^ Of note, the profiles of cytokine levels, including IL-6, IL-8, and IL-18, could be used to monitor the disease activity of SLE.^103,104^ Intervention strategies include glucocorticoids, antimalarial agents, nonsteroidal anti-inflammatory drugs, immunosuppressive agents, and B-cell-targeting biologics.^102^ Furthermore, CD19-targeted CAR-T cells also have the potential to eliminate B cells and control SLE.^105^ Other autoimmune diseases, such as autoimmune pancreatitis (AIP), psoriasis, rheumatoid arthritis, systemic vasculitis and ulcerative colitis, also show different patterns of cytokine elevation and different degrees of fever during disease progression. AIP is associated with increased production of type I IFN and is characterized by elevated IFN-I-dependent IL-33 production, which leads to fibrotic and inflammation responses.^106^ Psoriasis, an immune-mediated chronic disease, is pathogenically driven by pro-inflammatory cytokines, in which the IL-23/IL-17A axis plays the critical important role in pathogenesis.^107^

In summary, germline mutations in genes regulating the granule-mediated cytotoxicity, innate immune system, and inflammasome activation were the leading causes of autoinflammatory diseases and autoimmune diseases triggering CRS, while CD8 T cells and innate cells are most likely the primary driver cells, which produce and release TNF, IFN-γ, IL-1, and a combination of other cytokines to drive pathogenesis of these diseases.^1^

## Cellular mechanisms of CRS

### Cellular mechanisms of CRS in immunotherapy

**Fig. 3:**
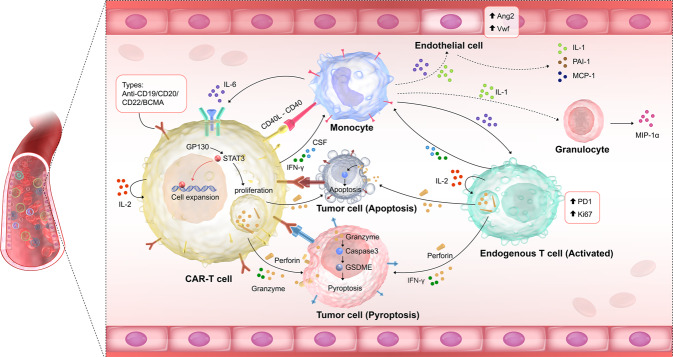
Cellular mechanisms of CAR-T-cell therapy triggering CRS. IL-6 *trans*-signaling promoted the expansion and antitumor activity of CAR-T cells via the GP130/STAT3 pathway, while apoptosis and pyroptosis were found in tumor cells after CAR-T-cell therapy; monocytes, endogenous T cells, endothelial cells, and granulocyte were all activated

CRS usually occurs at 1–10 days after CAR-T-cell infusion, accompanied by CAR-T-cell expansion and exhaustion. CAR-T cells and other cell subtypes are important in CAR-T-cell immunotherapy. In this section, we summarized new developments related to different cell types, levels (in vivo/in vitro levels; transcriptomic, epigenomic, and proteomic levels; and different species), dynamic changes, and potential interactions in CRS. Based on the interactions of CAR-T cells, endogenous T cells, monocytes, and endothelial cells, we proposed potential mechanisms of CRS in Fig. 3. The detailed information was provided as follows.

### Characterization and dynamics of CAR-T

Xue et al. performed single-cell multiplexed proteomic profiling of CD19 CAR-T-cell products from four patients in vitro with a 16-plex cytokine microfluidics device in 2017. They revealed that CAR-T cells showed high polyfunctionality in antitumor effector and stimulatory functions.^22^ In 2019, Xhangolli et al. demonstrated that CAR-T-cell activation involved a mixed T helper 1/2 cell response independent of differentiation in vitro.^108^ In their studies, both CD4 and CD8 CAR-T cells were equally effective in directly killing target tumor cells in vitro, and cytotoxic activity was associated with elevated coproduction of a wide range of cytokines (IFN-γ, TNF-α, GM-CSF, IL-5, IL-8, and IL-13). Additionally, cytotoxic T-lymphocyte-associated protein 4 (CTLA4) was the most upregulated immune checkpoint gene upon CAR-T-cell activation, which further correlated with the upregulation of IL-10 expression in the CD4 subset and that of TGFB1 in the CD8 subset.

Furthermore, Sheih et al. showed that the clonal diversity of CAR-T cells was highest in CAR-T-cell IPs and declined following infusion in vivo.^109^ Moreover, the clones that expanded after CAR-T infusion mainly originated from infused clusters with relatively high expression of cytotoxicity- and proliferation-related genes (CCL4, CD27, IFN-γ, GZMH, and GZMK). Furthermore, compared with the IPs, CD8 CAR-T cells at later times after infusion expressed higher proportions of inhibitory receptors (PD-1, LAG-3, TIM-3, KLRG1, TIGIT, 2B4, and CD160). At late and very late time points after CAR-T-cell infusion, the proliferative ability of CAR-T cells was decreased (decreasing gene expression of MKi67), which was accompanied by depletion of the target antigen.

### Characterization and dynamics of endogenous T cells

Our group longitudinally profiled the transcriptome of T cells in a plasma cell leukemia patient treated with anti-BCMA CAR-T cells and found that CAR-T cells in the CRS peak phase transition from a proliferative to a cytotoxic intermediate state, while the expression of GZMB and PRF1 was observed in endogenous T cells at the CRS peak phase.^110^ Meanwhile, in 2020, Chen et al. found that CAR-T cells composed only 1–5% of the total T-cell population within the intact tumor microenvironment (TME) in diffuse large B-cell lymphoma patients following CAR-T-cell therapy.^111^ Unexpectedly, a large number of non-CAR-T cells (endogenous T cells) within the TME were activated, and these cells were positive for Ki-67, IFN-γ, GZMB, and/or PD-1 and associated with beneficial and pathological effects.

### Characterization and dynamics of monocytes and macrophages

In 2018, Norelli et al. found that monocytes accumulated concomitantly with leukemia clearance in a humanized mouse CRS model.^29^ In detail, the counts of CD14 monocytes increased from day 1 to day 4 after CAR-T-cell infusion. Then, they started to decline and reached a minimum value at 7 days after CAR-T-cell infusion. Next, the counts of CD14 monocytes significantly increased and were maintained at a relatively high level during CRS recovery. Furthermore, monocyte ablation protected mice from CRS. These results suggested that CRS severity correlated with monocyte counts. In addition, the critical cytokines IL-6 and IL-1 were found to be mainly produced by monocytes during CRS, while the concentration of IL-1β in the serum increased approximately 24 h earlier than that of IL-6, and the level of IL-1β in the serum was lower than that of IL-6 after CAR-T-cell infusion. Another study also showed a similar phenomenon for monocytes. Giavridis et al. demonstrated that CRS severity was mediated not by CAR-T-cell-derived cytokines but by IL-6, IL-1, and nitric oxide (NO) produced by recipient macrophages (especially in the mouse peritoneum).^27^ Furthermore, they concluded that myeloid activation required proximity between CAR-T cells (CD40L) and macrophages (CD40), and indirect cytokine contact.

### Characterization and dynamics of endothelial cells

In 2017, Hay et al. demonstrated that severe CRS was accompanied by endothelial activation, in which the biomarkers of endothelial activation (angiopoietin-2 and von Willebrand factor) increased significantly.^34^ Additionally, the presence of vascular leakage and disseminated intravascular coagulation in patients with severe CRS was consistent with widespread endothelial activation.

### CRS mechanisms based on the interactions of different cell types

The pathways involved in severe CRS are mainly enriched in myeloid cell differentiation, cytokine production, and acute inflammatory response processes.^112^ We speculated that the mechanisms of CRS involved the interactions of CAR-T cells, monocytes, macrophages, endothelial and tumor cells, which included direct contact and indirect interactions. In 2018, Norelli et al.^29^ and Giavridis et al.^27^ reported that activation of monocytes and macrophages was a major contributor to the ‘amplification’ of the inflammatory response in CAR-T-cell therapy. Direct contact between CAR-T cells and monocytes/macrophages plays an important role in the activation of monocytes/macrophages. For example, CD40-CD40LG, CD69, lymphocyte activation gene 3 (LAG3), and membrane-expressed TNF-α have been demonstrated to activate monocytes/macrophages through contact-dependent mechanisms.^27^

On the other hand, indirect interactions (cytokine interactions) also play significant roles in CAR-T-cell-driven CRS. Among these cytokine interactions, CAR-T-cell-derived IL-6 is one of the most important initiators that amplifies the release of IL-6 from monocytes and further drives the development of severe CRS. In 2019, Jiang et al. revealed that IL-6 *trans*-signaling promoted the expansion and antitumor activity of CAR-T cells via the GP130/STAT3 pathway.^113^ Furthermore, in 2020, Kang et al. found that IL-6 knockdown in CAR-T cells via shRNA technology significantly reduced IL-6 release from monocytes in vitro.^114^ In addition to IL-6, the IL-1 and CSF pathways also play significant roles in CAR-T-cell-driven CRS via indirect interactions. For example, GM-CSF inactivation in CAR-T cells by TALEN-mediated genetic engineering prevents monocyte-dependent release of key CRS mediators in vitro.^23^ Then, our group showed significant interactions between highly cytotoxic CAR-T cells and endogenous T cells through the ligand–receptor pairs of CCR5-CCL5, CCL3-CCR5, CD70-CD27, and ICAM1-SPN/ITGAL.^110^ In addition to the interactions among CAR-T cells, monocytes and endogenous T cells, the crosstalk between CAR-T cells and tumors has also been studied. In 2020, Liu et al. demonstrated that CAR-T cells released GZMB, which activated caspase 3 to cleave GSDME in target tumor cells and caused pyroptosis. GSDME further leads to activation of caspase 1 and GSDMD in macrophages, which is critical for triggering CRS.^115^

### Cellular mechanisms of CRS in COVID-19

**Fig. 4:**
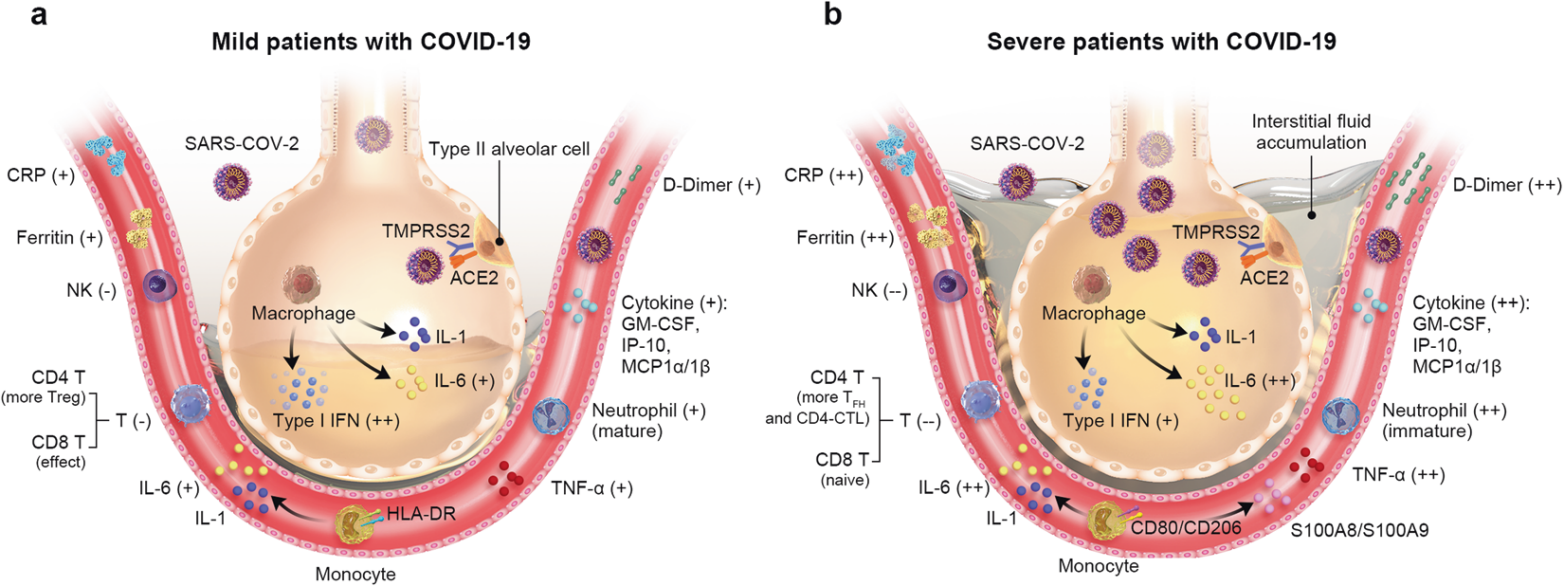
Cellular mechanisms of CRS in mild and severe patients with COVID-19. Mild patients have more NK and T cells in the peripheral blood (**a**), while severe patients are exposed to more COVID-19 and have more interstitial fluid accumulation (**b**). Neutrophil, C-reactive protein (CRP), ferritin, IL-6, TNF-α, D-dimer, and other cytokine in severe patients with COVID-19 were higher than that in mild patients

Understanding immune responses at the cellular and molecular level in COVID-19 helps to elucidate the mechanisms of host responses and interpret disease pathogenesis.^116^ Circulating T and B lymphocytes decreased significantly in COVID-19 patients, particularly in severe and critical stages of COVID-19 infection.^117^ Of note, such lymphopenia in patients with severe COVID-19 frequently occurs accompanying abnormal activation of monocytes/macrophages and an increase in neutrophil numbers.^118^ Other cells including NK and endothelial cells have also been reported to play an indispensable role in COVID-19 disease. The characteristics and comparisons of cytokine and cell types between mild and severe patients with COVID-19 were also shown in Fig. 4.

### Characterization and dynamics of T cells

Sekine et al. demonstrated that SARS-CoV-2-specific T cells were important for long-term immune protection against COVID-19,^119^ in which acute-phase SARS-CoV-2-specific T cells were highly activated and correlated with various clinical markers of disease severity, whereas convalescent-phase T cells were polyfunctional and displayed a stem-like memory phenotype. On the other hand, Zhu L et al. observed XIAP-associated factor 1 (XAF1)-, TNF-, and FAS-induced T cell apoptosis in COVID-19 patients.^120^

CD8 T resident memory and CD4 T helper 17 cells in mild COVID-19 undergo active expansion towards the end of the trajectory and are characterized by good effector functions, while in critical COVID-19 these remain more naive.^121^ Also, Meckiff et al. analyzed CD4 T cells from 40 COVID-19 patients, and showed that hospitalization (severe patients with COVID-19) is associated with increased cytotoxic follicular helper cells and cytotoxic T helper cells and a reduction in regulatory T cells.^122^ Moreover, in severe patients, the immune landscape featured a deranged IFN response, profound immune exhaustion with skewed T-cell receptor repertoire, and broad T cell expansion. Intensive expansion of highly cytotoxic effector T-cell subsets, such as granulysin (GNLY)^+^ CD4^+^ effector T cells, GNLY^+^CD8^+^ effector T cells and CD160 NKT cells, was associated with convalescence in moderate patients.^123^

### Characterization and dynamics of NK cells

It was reported that NK cells in the peripheral blood (PB) decreased in patients infected with SARS-CoV-2, especially in severe cases.^124^ However, another detached study showed no difference in the number of CD16^+^CD56^+^ NK cells in mild versus severe cases.^125^ On the other hand, Maucourant et al. illustrated a detailed NK cell activation landscape in bronchoalveolar lavage from COVID-19 patients^126^ and identified the hallmarks of these immunotypes, including perforin, NKG2C, and KSP37. They also observed arming of CD56 bright NK cells was driven by a defined protein–protein interaction network of inflammatory soluble factors in COVID-19.^126^

### Characterization and dynamics of monocytes and macrophages

Compared with normal healthy individuals, monocytes in PB from COVID-19 patients show an activated phenotype and secrete more IL-6, IL-10, and TNF-α,^127^ while the number of monocytes shows no significant difference. These activated monocytes in COVID-19 patients show features of mixed M1/M2 macrophage polarization, which is particularly associated with disease severity and poor prognosis. Besides, a large number of CD14^+^IL-1β^+^-activated monocytes were increased in COVID-19 patients.^117^ Furthermore, it was predicted that TNFSF13, IL-18, IL-2, and IL-4 may help to recover COVID-19 patients. Also, Guo Chuang et al. revealed a monocyte-associated CRS by single-cell sequencing.^128^ Lee et al. found that type I IFN response co-existed with the TNF/IL-1β-driven inflammation in classical monocytes from patients with severe COVID-19, while this was not seen in patients with milder COVID-19.^129^

Monocyte-to-macrophage trajectories showed that chronic hyperinflammatory monocytes accumulated, while anti-inflammatory and antigen-presenting macrophages in alveoli were depleted in critical COVID-19. Besides, monocytes may lead to fibrosis and worsen disease severity by ATP purinergic signaling.

### Characterization and dynamics of neutrophils

Neutrophils in circulation gradually increase as COVID-19 progresses and play a vital role in pathophysiology, particularly in patients with severe disease courses.^130^ In severe COVID-19 patients, dysfunctional neutrophils were associated with emergency myelopoiesis.^131^ Guo Qirui et al. found that coronavirus infection induced pathological damage by producing a certain group of neutrophils.^132^ Zhang et al.^133^ reported that the neutrophil-to-lymphocyte ratio (NLR) combined with IgG also helped to predict the severity of COVID-19.

Moreover, some studies have explored the function of neutrophil extracellular traps (NETs). Middleton et al. revealed that COVID-19 plasma would trigger NET formation by neutrophils and may lead to prothrombotic clinical presentations in COVID-19 patients.^134^ In addition, Barnes et al. also showed lung infiltration of neutrophils in an autopsy specimen from a COVID-19 patient and proposed that the ability to form NETs may contribute to organ damage and mortality in COVID-19.^135^

In conclusion, compared with mild patients, severe patients with COVID-19 are affected by higher load of SARS-CoV-2 at first, through the viral receptor ACE2 for entry into the cell and the serine protease TMPRSS2 for viral spike protein priming. Secondly, the pulmonary interstitial fluid accumulation and low type I IFN were observed in severe patients when compared with mild patients. Then, the cytokines (including CRP, Ferritin, IL-6, TNF-α, GM-CSF, IP-10, and MCP1α/1β) in the serum of severe patients with COVID-19 were higher than that in the mild patients with COVID-19, while the number of T and NK cells decreased and the number of neutrophils increased. In addition, the CD8 T cells and neutrophils in severe patients were of naive state, while these in mild patients were effective and mature. Finally, CD80/CD206 monocyte and their release factors S100A8 and S100A9 are positively associated with the severity of COVID-19, while HLA-DR monocyte is negatively associated with the severity of COVID-19.^136^

### Molecular mechanisms of CRS

According to the important cytokines of CRS mentioned above, the present review illustrated the possible molecular and cellular mechanisms of CRS from several key cytokine-triggered signaling pathways, including TNF/NF-κB, IL-1/NF-κB, IL-6/JAK-STAT, and IFN-γ/JAK/STAT signaling pathways.

### TNF/NF-κB signaling pathways

TNF-α is a well-known pro-inflammatory cytokine, mainly produced by monocytes and T cells and is involved in early inflammatory events. As an initial driver of nuclear factor κB (NF-κB) signaling pathway, TNF-α can induce the expression of a variety of anti-apoptotic and pro-inflammatory genes by activating its receptor TNFR1 and intermediate adapters. NF-κB plays critical roles in the immune system, particularly in regulating the expression of some inflammation-related cytokines. Hence, NF-κB in turn can induce TNF-α expression.^137^

TNF-α is closely associated with iatrogenic diseases, pathogenic diseases, and monogenic and autoimmune diseases. TNF-α blockade (adalimumab) exerted separated and synergistic effects on preventing endothelial activation induced by CAR-T, myeloid cells, and tumor cells.^138^ Besides, adalimumab prevents macrophage activation and effectively mitigates CRS in anti-CD3 bispecific antibody therapy.^61^ Furthermore, in SARS-CoV and MERS cases, excessive TNF-α represented a poor prognosis and inhibition of NF-κB can increase survival in SARS-CoV-infected mice.^139^ Moreover, deregulation of TNF-α characterizes many autoimmune diseases.^140^ Therefore, TNFα/NF-κB signaling may play pathological roles in the initiation of CRS and the over-activation of immune cells during CRS.

### IL-1/NF-κB signaling pathways

IL-1 family cytokines play a vital role in immune systems and mediate inflammation in response to various stimuli. From the cellular level, IL-1 is mainly secreted by monocytes, macrophages and dendritic cells, which were encoded by IL1A and IL1B.^29,141^ Then, IL-1β from activated myeloid cells further induced endothelial activation and neutrophil production. On the other hand, the basic molecular mechanism of IL-1/NF-κB signal initiation is a stepwise process: (1) IL-1β was formed from inactivate IL-1β precursors by cleavage of NLRP3 inflammasomes. (2) IL-1α/β bind to the same IL-1 receptor, activating a cascade of intracellular NF-κB.

In CAR-T therapy, CRS severity correlated with monocyte/macrophage counts and critical cytokines IL-1 and IL-6 were mainly produced by monocytes during CRS. IL-1β may also contribute to CRS in coronavirus infections.^142^ In many autoinflammatory disorders, IL-1 is the critical player of the innate immune response and inflammation.^143^ Anti-IL1β antibody exerted separated and synergistic effects in preventing endothelial activation induced by tumor cells, CAR-T, and myeloid cells.^138^ In severe COVID-19, it was proposed that NLRP3 activation and IL-1β precursor cleavage potentially aggravate CRS.^144^

### IL-6/JAK-STAT signaling pathways

IL-6 is an inflammation-related cytokines and activates the JAK/STAT3 pathway by deploying *cis*-signaling and *trans*-signaling. In *cis*-signaling, IL-6 binds to membrane-bound IL-6R that was restrictedly expressed on immune cells, and then combined with gp130 and activated downstream JAK/STAT3 and other signaling pathways. In *trans*-signaling, circulating IL-6 firstly binds to soluble IL-6Rs and then binds with the gp130 dimer which was expressed on almost all cell subpopulation. This *trans*-signaling of the JAK–STAT3 pathway expanded inflammation from immune cells to cells without mIL-6R expression, such as endothelial cells and vascular smooth muscle cells. The crushing activation of the IL-6-IL-6R/JAK-STAT3 signaling results in systemic hyperinflammation and provokes the secretion of various mediators.^145^ These results may further contribute to vascular hyperpermeability, leakiness, hypotension, and pulmonary dysfunction during CRS.

IL-6 has been shown to be markedly elevated in patients with multiple CRS. Besides CAR-T-induced CRS in adults and children,^29,146^ many studies also showed that the serum of IL-6 increased remarkably in patients with COVID-19.^147–149^ Besides, targeting IL-6 pathway has led to innovative therapeutic approaches for various rheumatic diseases and related therapy is on the way.

### IFN-γ/JAK/STAT signaling pathways

IFN-γ is a potent activator of macrophages, mainly secreted by NK cells and activated T cells.^150^ IFN-γ is involved in inflammation and other immunological processes, which plays a major role in protective immunity against bacterial and viral infections by activating JAK1/STAT cascades. More specifically, IFN-γ activates JAK/STAT-associated signaling pathway by binding to its two receptor-subtypes (IFN-γ receptor (IFNGR)-1 and -2), and achieve transcriptional activation of IFN-γ-inducible genes. The major STAT protein activated by IFN-γ is STAT1 and the IFN-γ-inducible genes are IFN regulatory factor (IRF)-1, CXCL10, CXCL11, and IFIT2.^151,152^

Different studies have demonstrated that IFN-γ is highly involved in a variety of CRS-related diseases. In CAR-T therapy, the production of IFN-γ was mediated by myeloid-derived macrophages and plays a critical role in CRS pathogenesis.^153^ Elevated levels of the above core cytokines have been confirmed in patients in clinical^146^ and in murine models.^32^ In primary HLH, large quantities of IFN-γ were produced and excessive T cells were activated.^154^ In COVID-19, numerous studies have reported elevated levels of IFN-γ in patients, which may mainly from macrophages, not T cells.

**Fig. 5:**
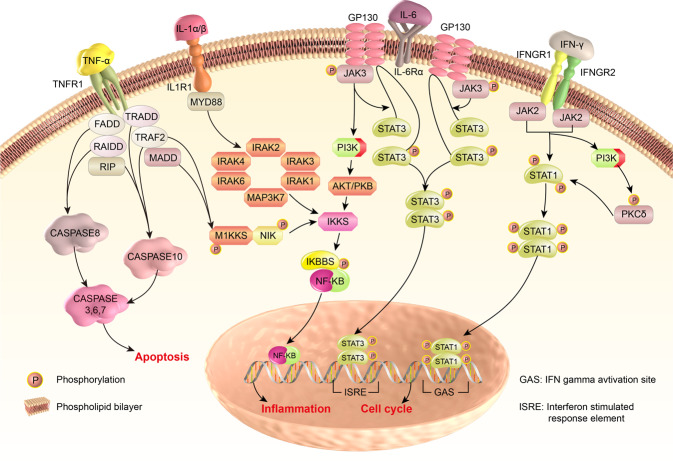
Molecular mechanisms of CRS from TNF/NF-κB, IL-1/NF-κB, IL-6/JAK-STAT, and INF-γ/JAK-STAT pathway

To further strengthen the understanding of CRS, the molecular mechanisms of CRS were summarized from TNF/NF-κB, IL-1/NF-κB, IL-6/JAK-STAT, and INF-γ/JAK-STAT pathways and shown in Fig. 5.

## CRS models

### CRS models in CAR-T-cell immunotherapy

CRS models are essential for the analysis of CRS mechanisms. In this section, we summarize the detailed methods of CRS mouse model studies, including mouse models (mouse strain, hematopoietic stem cells (HSCs), leukemia cells, CAR-T cells, and injection methods), cytokine detection panels, time points, and weight and fever information.

In the severe combined immunodeficiency (SCID)-beige mouse model, Sterner et al. found that GM-CSF inhibition not only reduced CRS severity but also enhanced CAR-T-cell functions in xenografts.^24^ Furthermore, compared with CD19 CAR-T cells, GM-CSF-deficient CD19 CAR-T cells maintained normal functions and enhanced antitumor activity and improved overall survival. In another mouse CRS model study, Mestermann et al. demonstrated that a short treatment course of dasatinib protected a subset of mice from lethal CRS, indicating that this treatment may be a broadly applicable pharmacologic on/off switch for CAR-T cells.^155^

The mouse myeloid system (monocytes and macrophages) in the SCID-beige mouse model is not well matched with human CAR-T cells killing leukemia cells. Therefore, the humanized NSG mouse model is more widely used in different studies.^29,156,157^ In 2017, Diaconu et al. demonstrated that in a humanized mouse model, the inducible caspase-9 (iC9) safety switch could eliminate CD19 CAR-T cells in a dose-dependent manner, allowing either selective containment of CD19 CAR-T-cell expansion in the case of CRS or complete deletion on demand to allow normal B-cell reconstitution.^156^ In 2018, by using a humanized NSG mouse model, Norelli et al. demonstrated that CRS was characterized by high fever and elevated IL-6 levels and that the major source of IL-1 and IL-6 during CRS is monocytes.^29^ In addition, in 2020, Sun et al. revealed that the kinase LCK and phosphatase SHP1 can be engineered in both CD28 and 4-1BB CAR-T cells to tune CAR-T-cell activity by affecting the magnitude of CAR-T-cell activation. In their humanized mouse model, SHP1 recruitment induced by the transient administration of AP21967 alleviated weight loss and significantly reduced the release of human IFN-γ, IL-6, GM-CSF, and TNF-α into the plasma.^157^

**Fig. 6:**
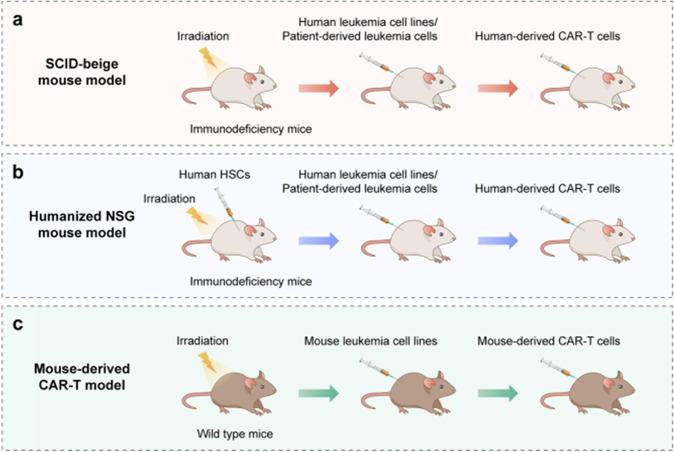
CRS mouse models. **a** Severe combined immunodeficiency (SCID)-beige mouse model. **b** Humanized NSG mouse model. **c** Mouse-derived CAR-T model

In addition to the above models, there is another mouse-derived CAR-T model in which mouse CAR-T cells recognize mouse leukemia cells in the mouse microenvironment, which would be beneficial for exploration of CRS mechanisms (Fig. 6).

### CRS model in COVID-19

Animal models of SARS-CoV-2 infection are important and urgently needed to improve research and drug/vaccine development. In 2020, the Qin Chua’s group established animal models of SARS-CoV-2 in transgenic mice that express human ACE2 (hACE2 mice). Weight loss, virus replication, viral antigens, and interstitial pneumonia were observed in hACE2 mice after SARS-CoV-2 infection, while these phenomena were not found in wild-type mice infected with SARS-CoV-2.^158^ In addition, the Shi Zheng-Li’s group developed a mouse model with symptoms similar to those of patients with COVID-19 by transgenic technology (HFH4-hACE2 in C3B6 mice).^159^ In the HFH4-hACE2 COVID-19 model, HFH4was used to control human ACE2 expression. In addition to ACE2, other genes (such as TMRPSS2 and FcγRT) are also critical for COVID-19 infection. Therefore, multiple humanized COVID-19 models will be a direction for future study.

## Cutting-edge technologies in CRS-related studies

### Single-cell technologies

#### Single-cell RNA sequencing

Single-cell RNA sequencing, especially 10× genomic single-cell transcriptome sequencing, has become a powerful tool for revealing the dynamic changes in various subpopulations and the source of key cytokine both in the CAR-T-cell therapy and COVID-19.^129,136,160–162^ The sample types and time points of the above single-cell studies on CAR-T- and COVID-19-induced CRS were summarized in Table 4. Single-cell studies of CAR-T mainly focus on the changes of CAR-T cells, while the single-cell studies of COVID-19 mainly focus on the comparison of severe patients, mild patients, and healthy donors among different tissues, especially the lung and PB.

**Table 4:** Single-cell studies on CAR-T-cell therapy and COVID-19

Cell type	Sample/Time	Conclusions
CAR-T-cell therapy		
CAR-T cells	CRS initiation stage	CD8 41-BBz CAR-T enriched in central memory cell phenotype and fatty acid metabolism genes (Boroughs et al., 2020)^163^
CAR-T cells	CRS initiation stage	CD4 helper and CD8 cytotoxic CAR-T cells are equally effective in directly killing tumor cells (Xhangolli et al., 2019)^108^
Leukocyte	CRS peak stage	High fever and elevated IL-6 levels are hallmarks of CRS; Human monocytes are the major source of IL-1 and IL-6 during CRS (Norelli et al., 2018)^29^
CAR-T and endogenous T	CRS peak stage	CAR-T cells in the CRS peak phase transition from a proliferative to a cytotoxic intermediate state; Endogenous T presents an active state in the CRS peak stage (Li et al., 2021)^110^
CAR-T cells	CRS recovery stage	Clonal diversity of CAR-T cells is highest in products and declines following infusion; Amplified oligoclones at last mainly originate from CAR-T products with high expression of cytotoxicity and proliferation genes (Sheih et al., 2020)^109^
CAR-T cells	CRS recovery stage	Heterogeneity of CAR-T cells contributes to variations in efficacy and toxicity in LBCL (Deng et al., 2020)^164^
COVID-19		
PBMC	76 COVID-19 patients and 69 healthy donors	Enhanced plasma levels of inflammatory mediators, including EN-RAGE, TNFSF14, and oncostatin M, were correlated with COVID-19 severity (Arunachalam et al., 2020)^160^
PBMC	3 COVID-19 patients and 3 healthy donors	Immature neutrophil and nonclassical monocyte pools, with levels of the protein calprotectin linked to disease severity (Silvin et al., 2020)^161^
PBMC	9 COVID-19 patients, 5 influenza patients and 4 healthy donors	TNF/IL-1β–driven inflammation was defining characteristics of COVID-19.The type I IFN response plays a pivotal role in exacerbating inflammation in severe COVID-19 (Lee et al., 2020)^129^
PBMC	40 COVID-19 patients and 13 healthy donors	Hospitalization is associated with increased cytotoxic follicular helper cells and cytotoxic T helper cells and a reduction in regulatory T cells (Meckiff et al., 2020)^122^
BALF	6 Severe- and 3 mild- COVID-19 patients	Dramatic differences between the mild and severe COVID-19 patients, including an inflammatory signature. SARS-CoV-2 infects epithelial cells and alters immune landscape in severe patients (Bost et al., 2020)^123^
PBMC	5 COVID-19 patients, 2 influenza virus patients and 2 healthy donors	COVID-19 patient features XAF1-, TNF-, and FAS-induced T cell apoptosis. COVID-19 activates distinct pathway (STAT1/IRF3) versus influenza, and substantial differences were revealed like expression of interleukin (IL)6R and IL6ST (Zhu L et al., 2020)^120^
PBMC	2 COVID-19 patients and 2 healthy donors	A monocyte subpopulation contributes to the inflammatory CRS and tocilizumab treatment can attenuate the CRS in COVID-19 patients (Guo et al., 2020)^128^
PBMC and whole blood	53 COVID-19 patients and 56 healthy donors	HLA-DR^hi^CD11c^hi^ inflammatory monocytes with an IFN-stimulated gene signature were elevated in mild COVID-19. Severe COVID-19 was marked by the occurrence of neutrophil precursors (Schulte-Schrepping et al., 2020)^131^
PBMC	13 COVID-19 patients and 5 healthy donors	COVID-19 showed a strong IFN-α response and an overall acute inflammatory response. In severe patients, the immune landscape featured a deranged interferon response (Zhang et al., 2020)^133^
PBMC	7 Severe COVID-19 patients and 6 healthy donors	Severe disease has been associated with changes in peripheral immune activity, including increased levels of pro-inflammatory cytokines, while peripheral monocytes and lymphocytes do not express substantial amounts of pro-inflammatory cytokines (Wilk et al., 2020)^162^
PBMC, BALF/PFMC, sputum	196 COVID-19 patients and 25 healthy donors	Systemic upregulation of S100A8/A9, mainly from megakaryocytes and monocytes in the peripheral blood, may contribute to the CRS frequently in severe patients (Ren et al., 2021)^136^

In addition to the above single-cell studies, Boroughs et al.^163^ profiled the transcriptome of CAR-T cells in vitro and found that the differentially expressed genes of 4-1BBz CAR CD8 T cells were enriched in central memory cell phenotype and fatty acid metabolism genes compared to that of 28z CAR-T cells. Meanwhile, Deng et al. performed single-cell RNA sequencing of anti-CD19 CAR-T-cell IPs in 24 patients with LBCL. Apart from finding that a rare cell population with monocyte-like transcriptional features in CAR-T products was associated with high-grade ICANS, they also observed that high-grade CRS was negatively correlated with exhausted CD8 T cells and positively correlated with exhausted CD4 T cells.^164^

#### Single-cell TCR and ATAC sequencing

In addition to scRNA-seq, single-cell T-cell receptor (TCR) sequencing and single-cell assays for transposase-accessible chromatin using sequencing (scATAC-seq) have also been employed in studies of CAR-T-cell expansion. Sheih et al.^109^ described the alterations of clonal diversity of CAR-T cells during the course of the disease by single-cell TCR sequencing. To be specific, the clonal diversity of CAR-T cells was highest in the IPs and declined following infusion. For COVID-19, Nguyen et al. analyzed ex vivo CD8+ T cells specific for COVID-19 epitopes and found that B7/N105+CD8+ T cells were seen in high numbers during COVID19 and persisted for a long time, by the integrative analysis of high naive frequency and TCR plasticity.^165^

Using scATAC-seq, Wang et al. showed that the precise location of the CAR vector in the patient genome may play an essential role in treatment outcomes.^166^ Combined with bulk and single-cell ATAC sequencing, Chen et al. found that IRF7 could affect CAR-T-cell persistence across T-cell subsets by regulating chronic IFN signaling, and that the TCF7 regulon is maintained in effector T cells among patients with long-term CAR-T-cell persistence.^167^ The dynamic epigenetic changes in various cell types during the CRS process are also worthy of further study.

#### Single-cell multiplexed cytokine assay

A multiplexed single-cell cytokine assay method has been employed to identify and predict CRS biomarkers after CAR-T-cell therapy. In 2016, Teachey et al. found that the expression levels of 24 cytokines were highly associated with different CRS grades in CAR-T-cell therapy by using the above technology. Furthermore, prediction models based on IFN-γ/sgp130/sIL-1RA expression levels could accurately predict which patients would develop severe CRS.^168^ For COVID-19, Mathew et al. analyzed 125 COVID-19 patients at the protein level by high-dimensional flow cytometry and identified three prominent and distinct immunotypes that are related to disease severity and clinical parameters.^169^ Among them, immunotypes with robust activated CD4 T cells, activated CD8 T cells, and plasmablasts were associated with disease severity of COVID-19.

#### Other cutting-edge technologies

Gene editing is another new advanced technology and the introduction of gene editing into CAR-T cells would be a new direction for preventing severe CRS. Kang et al. found that IL-6 knockdown of CAR-T cells significantly reduced IL-6 release from monocytes in vitro.^114^ Drug-screening is also an effective technology to temporarily switch off CAR-T cells. In a mouse model of CRS, Mestermann et al. found that dasatinib halted cytokine production and proliferation of CAR-T cells and protected mice from otherwise fatal CRS, while the inhibitory effect was rapidly and completely reversed upon discontinuation of dasatinib.^155^ In addition, Sun et al. found that SHP1 recruitment induced by the transient administration of AP21967 significantly reduced the release of human IFN-γ, IL-6, GM-CSF, and TNF-α in the plasma of a humanized mouse model.^157^ At last, novel types of CAR are also a direction to fundamentally prevent CRS. For example, CAR NK cells can induce responses in patients with r/r CD19-positive cancers without severe CRS.^170^

#### Management of CRS by intervention targeting CRS-related pathways

CRS management is essential for CAR-T-cell therapy and patients with COVID-19 and monogenic and autoimmune diseases. Here, we summarized drugs targeting specific cytokines and overlapping signaling pathways for intervening.

#### Drugs targeting TNF/NF-κB signaling pathways

TNF, a key upstream factor mediating monocyte activation, can result in inflammation, fever, and cellular proliferation. The TNF–TNF receptor superfamily can lead to the expression of multiple pro-inflammatory genes by inducing NF-κB. In anti-CD3 bispecific antibody therapy, adalimumab (anti-TNF-α drug) prevents macrophage activation and effectively mitigates CRS.^61^ Furthermore, the effect of adalimumab on COVID-19 patients has also been investigated.^171^

#### Drugs targeting IL-1/NF-κB signaling pathways

IL-1 activates a cascade involving intracellular nuclear factor κB (NF-κB) and other significant pathways. The IL-1R antagonist anakinra is effective for the treatment of CRS in CAR-T-cell therapy,^29^ secondary HLH,^172^ and COVID-19.^173,174^

#### Drugs targeting IL-6/JAK-STAT signaling pathways

IL-6 plays a vital role in acute inflammatory response and CRS and is highly elevated in CAR-T-cell therapy patients, COVID-19 patients, and monogenic and autoimmune diseases.^175^ Targeted IL-6 receptor drug (tocilizumab) and neutralizing IL-6 drug (siltuximab) have already been in clinical use to treat CRS disorders, including HLH- and CAR-T-cell-induced CRS.^146^ In addition, in severe and critical COVID-19 patients, the Wei Haiming’s group suggested that tocilizumab improved clinical outcomes immediately and was an effective treatment to reduce mortality.^176^ However, in moderately ill patients with COVID-19, tocilizumab was not effective for preventing mechanical ventilation or death.^177^

#### Drugs targeting IFN-γ/JAK/STAT signaling pathways

IFN-γ, a potent activator of macrophages is primarily secreted by activated T and NK cells. The US FDA approved anti-IFN-γ antibody emapalumab have been used to treat patients with relapsed/refractory HLH, based on favorable results in a single-arm, open-label Phase II/III trial (NCT01818492; NCT02069899).^178^ In addition, neutralizing antibodies against IFN-γ improved animal survival in a mouse model of LPS-induced sepsis.^179^ Both interventions in animal models and clinical trials of therapies targeting the type I IFN signaling pathway have shown efficacy in the treatment of autoimmune diseases, such as SLE, rheumatoid arthritis, juvenile idiopathic arthritis, juvenile dermatomyositis, and systemic sclerosis.^180^ Furthermore, anti-IFN-γ antibody treatment improved survival and reduced pro-inflammatory cytokines in experimental model of macrophage activation syndrome associated with juvenile idiopathic arthritis.^181^

In summary, the most common clinical management approach for CRS after CAR-T-cell therapy is supportive care. When severe CRS occurs, drugs such as tocilizumab, siltuximab, or corticosteroids are always used. In CAR-T-cell therapy, anti-IL-6 drug therapy is recommended to treat hypotension and hypoxia for patients with a higher grade of CRS.^32^ When patients are refractory to anti-IL-6 therapy, the usage of corticosteroids^182,183^ can be considered to inhibit T cell activation by downregulating signal transduction through the IL-2 receptor. For the management of CRS in COVID-19, except for the above methods, some specific interventions or targets were provided, including early type I IFN response.^67^ Preliminary reports showed that IFN alpha-2b combined with LPV/r accelerated viral clearance in COVID-19 patients and shortened the length of hospitalization.^184^ In addition, paquinimod, a specific inhibitor of S100A8/A9, could significantly reduce viral load and the activation of aberrant neutrophils in SARS-CoV-2-infected mice, and rescue pneumonia.^132^ At last, as patients treated with CAR-T cell are intrinsically more susceptible to SARS-CoV-2 infection, our group recommend that high-dose steroids should be avoided because they could worsen CAR-T-cell therapy outcomes and disrupt CAR-T-cell persistence. Furthermore, tocilizumab or artificial liver treatment is recommended for severe COVID-19 patients with elevated cytokine levels.^185^

#### Perspectives

A subset of patients receiving CAR-T/antibodies or with COVID-19, monogenic and autoimmune diseases display severe CRS. In the present review, we summarized the clinical features, risk factors, cellular and molecular mechanisms, and management of CRS in the above human diseases, as well as CRS models and cutting-edge technologies in CRS studies.

The elevated serum cytokines in the patients with COVID-19, monogenic and autoimmune diseases or CAR-T/antibody treatment were similar (including IL-6, TNF, IFN-γ, CRP, and D-dimer), while signaling pathways involved in the regulation of CRS also overlapped (such as IL-1/NF-κB, TNF/NF-κB, IL-6/JAK-STAT, and INF-γ/JAK-STAT signaling pathway). In addition, the differences in CRS among phenotype and etiologies should also be noted. For example, lymphopenia (low blood lymphocyte count) is one of the key characteristics of severe COVID-19 patients,^186^ while lymphocytes (including CAR-T and endogenous T cells) are activated and increase in number in CAR-T-cell therapy.^110^ Our group also compared the clinical characteristics and risk factors associated with severe CRS induced by COVID-19 and CAR-T-cell therapy.^187^ We found a significantly higher incidence of grade 4 CRS in the COVID-19 group than in the CAR-T-cell therapy group. In addition, COVID-19 group showed increased TNF-α level and decreased IL-2, IL-6, IL-10, and IFN-γ levels.

Intervention therapy for CRS is also an important issue. Several strategies to diminish undesirable CRS were summarized: (a) drug intervention, including multitarget drugs, targeted mAb drugs, targeted monocyte-clearing drugs (liposomal clodronate,^29^) and small molecules; (b) the structure of the CAR-T vector, including new structure of CAR-T vector,^188^ novel CAR-NK cells, CAR-macrophage cells^189^ and dual-target CAR-T cells, and their infusion strategy and dose; (c) gene editing of CAR-T cells, including IL-6^114,190^ and GM-CSF^24^ knockout and naive memory-related gene (LPR6 targeting^191^) overexpression; and (d) low-molecular-weight adapters.^192^

In addition to CRS management, some unclear issues related to CRS and future directions are also summarized in the present review. For example, the crosstalk of endothelial cells, granulocytes, and CAR-T cells is worthy of further verification and exploration. Meanwhile, transcriptome-level data alone cannot solve all the problems of CAR-T-cell expansion and exhaustion issues.^193^ Therefore, in the future, single-cell multiomics and other new cutting-edge technologies, including CAR-T-cell integration site analysis, scRNA-seq, scATAC-seq, CyTOF and imaging technology, should be combined for the CRS studies.

CRS is a double-edged sword, which facilitates disease control and may be harmful to the host. Our goal is to illustrate the framework, characteristics and mechanism of CRS in human diseases and achieve both favorable efficacy and low toxicity.
