# Correction: Signaling pathways in the regulation of cytokine release syndrome in human diseases and intervention therapy

**DOI:** 10.1038/s41392-021-00815-w

**Published:** 2021-11-18

**Authors:** Xia Li, Mi Shao, Xiangjun Zeng, Pengxu Qian, He Huang

**Affiliations:** 1 Bone Marrow Transplantation Center, The First Affiliated Hospital, Zhejiang University School of Medicine, Hangzhou, People’s Republic of China; 2 Liangzhu Laboratory, Zhejiang University Medical Center, 1369 West Wenyi Road, Hangzhou, 311121 People’s Republic of China; 3 Institute of Hematology, Zhejiang University, Hangzhou, Zhejiang People’s Republic of China; 4 Zhejiang Province Engineering Laboratory for Stem Cell and Immunity Therapy, Hangzhou, Zhejiang People’s Republic of China; 5 Center of Stem Cell and Regenerative Medicine, Zhejiang University School of Medicine, Hangzhou, People’s Republic of China

**Keywords:** Infectious diseases, Immunotherapy, Immunological disorders

Correction to: *Signal Transduction and Targeted Therapy* 10.1038/s41392-021-00764-4, published online 20 October 2021

Since the publication of this review^1^, the authors noticed one inadvertent mistake occurred in Fig. 6c that needs to be corrected. The correct Fig. 6 is provided as follows. In detail, “Human-derived CAR-T cells” has been replaced by “Mouse-derived CAR-T cells” in the revised fig. 6c. The key findings of the article are not affected by these corrections.

**Fig. 6:**
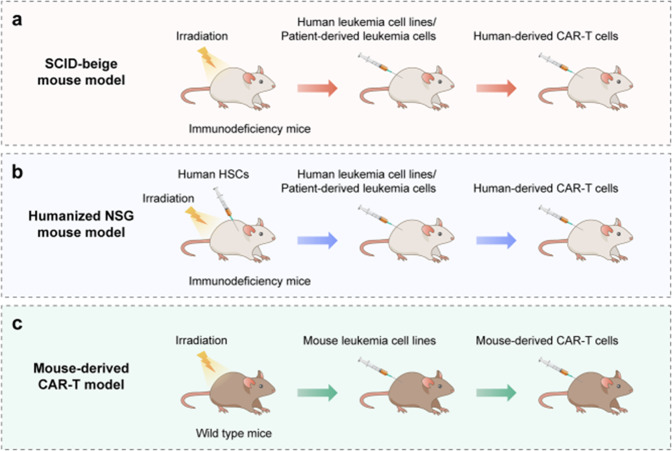
CRS mouse models. **a** Severe combined immunodeficiency (SCID)-beige mouse model. **b** Humanized NSG mouse model. **c** Mouse-derived CAR-T model

The original review article has been corrected.
